# Comment on “Improving consenting practice in trauma and orthopaedics: A single centre original mixed methods study”

**DOI:** 10.1016/j.sipas.2025.100325

**Published:** 2025-12-20

**Authors:** Ankit Batra, Prashant Kokiwar, Priyanka Aher, Indu Singh

**Affiliations:** a Department of Orthopedics, School of Medical Sciences and Research, Sharda University, Greater Noida, India; b Department of Community Medicine, Malla Reddy Institute of Medical Sciences, Malla Reddy Vishwavidyapeeth, Suraram, Hyderabad 500055, Telangana, India; c Dr. D. Y. Patil Medical College, Hospital and Research Centre, Dr. D. Y. Patil Vidyapeeth (Deemed-to-be-University), Pimpri, Pune 411018, Maharashtra, India; d Faculty of Pharmaceutical Sciences, Graphic Era Hill University, Dehradun, India; e Centre for Promotion of Research, Graphic Era Deemed University, Dehradun, India

**Keywords:** Informed consent, Electronic consent, Trauma and orthopaedics, Shared decision making

Dear Editor,

We read with interest the article titled, “Improving consenting practice in trauma and orthopaedics: A single centre original mixed methods study” [1]. The authors report frequent same-day consenting, illegible documentation, incomplete recording of risks and alternatives, and clinician support for electronic consent solutions. We agree that improving consent quality is a clinical and medico-legal priority and welcome their focus on pragmatic change in a district general hospital.

One conceptual issue is the assumption that better documentation equates to better patient understanding. The audit evaluates legibility and completeness of forms, but not comprehension, satisfaction or decisional conflict. Contemporary work on e-consent shows that digital tools can reduce scheduling delays yet many patients simply skim forms and still rely on surgeon discussion for meaning [2]. Incorporating patient-reported outcomes and brief knowledge assessments would align future cycles with shared decision-making standards [3].

There is also a risk of over-interpreting a small, single-centre dataset. Only 102 forms and 16 clinicians are included, with limited detail on case mix, urgency and sampling strategy, making external validity difficult to judge. A 40 % clinician survey response rate raises the possibility of selection bias. Describing non-responders and stratifying documentation quality by elective versus urgent procedures, as well as by timing of consent, would clarify where deficits are most pronounced.

Finally, while enthusiasm for e-consent is justified, the discussion could acknowledge uncertainties highlighted by recent syntheses. Electronic platforms consistently improve documentation and efficiency, but evidence for effects on equity, access and meaningful autonomy remains limited [4]. Normative guidance stresses that digital solutions should augment, not replace, iterative, relationship-based consent conversations and time for reflection. Within trauma and orthopaedics, pragmatic evaluation of procedure-specific digital consent co-designed with patients and informed by evidence that multimedia tools can enhance perioperative understanding would build directly on this important audit [5].

Addressing these points would strengthen interpretation of the findings and help ensure that future interventions genuinely improve patient-centred consent practice.

## CRediT authorship contribution statement

**Ankit Batra:** Conceptualization, Data curation, Formal analysis, Funding acquisition, Investigation, Methodology, Project administration, Resources, Software, Supervision, Validation, Visualization, Writing – original draft, Writing – review & editing. **Prashant Kokiwar:** Conceptualization, Data curation, Formal analysis, Funding acquisition, Investigation, Methodology, Project administration, Resources, Software, Supervision, Validation, Visualization, Writing – original draft, Writing – review & editing. **Priyanka Aher:** Conceptualization, Data curation, Formal analysis, Funding acquisition, Investigation, Methodology, Project administration, Resources, Software, Supervision, Validation, Visualization, Writing – original draft, Writing – review & editing. **Indu Singh:** Conceptualization, Data curation, Formal analysis, Funding acquisition, Investigation, Methodology, Project administration, Resources, Software, Supervision, Validation, Visualization, Writing – original draft, Writing – review & editing.

## Declaration of competing interest

The authors declare that they have no known competing financial interests or personal relationships that could have appeared to influence the work reported in this paper.

## References

[1] Jacob K.J., Mangal M.N., Lamb J.W.M., Abdallatif A., Mathur K., Elmajee M. (2025). Improving consenting practice in trauma and orthopaedics: a single centre original mixed methods study. Surg Pr Sci.

[2] Trang K., Decker H.C., Gonzalez A., Pierce L., Shui A.M., Melton-Meaux G.B. (2024). Electronic surgical consent delivery via patient portal to improve perioperative efficiency. JAMA Surg.

[3] St John E.R., Moore C.J.S., Pillarisetti R.R., Spatz E.S. (2024). Global considerations for informed consent with shared decision-making in the digital age. BMJ Evid-Based Med.

[4] Chimonas S., Lipitz-Snyderman A., Matsoukas K., Kuperman G. (2023). Electronic consent in clinical care: an international scoping review. BMJ Health Care Inf.

[5] Shepherd T., Trinder M., Theophilus M. (2022). Does virtual reality in the perioperative setting for patient education improve understanding? A scoping review. Surg Pr Sci.

